# Developing antibacterial peptides as a promising therapy for combating antibiotic-resistant *Pseudomonas aeruginosa* infections

**DOI:** 10.14202/vetworld.2024.1259-1264

**Published:** 2024-06-08

**Authors:** Rula M. Darwish, Ali H. Salama

**Affiliations:** 1 Department of Pharmaceutics and Pharmaceutical Technology, School of Pharmacy, the University of Jordan, Amman, 11942, Jordan; 2 Department of Pharmacy, Faculty of Pharmacy, Middle East University, Amman, 11831, Jordan

**Keywords:** antimicrobial resistance, peptides, *Pseudomonas aeruginosa*, synergism

## Abstract

**Background and Aim:**

Antibiotic-resistant *Pseudomonas aeruginosa* poses a serious health threat. This study aimed to investigate the antibacterial activity of peptide KW-23 against drug-resistant *P. aeruginosa* and its potential for enhancing the efficacy of conventional antibiotics.

**Materials and Methods:**

KW-23 was synthesized from nine amino acids, specifically three tryptophans and three lysines. The purity of the substance was analyzed using reverse-phase high-performance liquid chromatography. The peptide was identified through mass spectrometry using electrospray ionization. The minimum inhibitory concentration (MIC) values of KW-23 in combination with conventional antibiotics against control and multidrug-resistant *P. aeruginosa* were determined utilizing broth microdilution. The erythrocyte hemolytic assay was used to measure toxicity. The KW-23 effect was analyzed using the time-kill curve.

**Results:**

The peptide exhibited strong antibacterial activity against control and multidrug-resistant strains of *P. aeruginosa*, with MICs of 4.5 μg/mL and 20 μg/mL, respectively. At higher concentration of 100 μg/mL, KW-23 exhibited a low hemolytic impact, causing no more than 3% damage to red blood. The cytotoxicity assay demonstrates KW-23’s safety, while the time-kill curve highlights its rapid and sustained antibacterial activity. The combination of KW-23 and gentamicin exhibited synergistic activity against both susceptible and resistant *P. aeruginosa*, with fractional inhibitory concentration index values of 0.07 and 0.27, respectively.

**Conclusion:**

The KW-23 synthesized in the laboratory significantly combats antibiotic-resistant *P. aeruginosa*. Due to its strong antibacterial properties and low toxicity to cells, KW-23 is a promising alternative to traditional antibiotics in combating multidrug-resistant bacteria.

## Introduction

*Pseudomonas aeruginosa*, a globally prevalent Gram-negative bacterium, significantly influences human health as a potent opportunistic pathogen [1]. Known for its adaptability and resilience, *P. aeruginosa* is linked to various types of infections, from localized skin and soft-tissue infections to serious systemic diseases [2]. This bacterium is known for causing severe infections in immunocompromised patients, including those with cystic fibrosis, burn wounds, and undergoing invasive medical procedures [3]. *P. aeruginosa*’s strong biofilm formation and resistance acquisition make it a major clinical challenge, resulting in persistent infections that are hard to eliminate [4]. The growing antibiotic resistance in *P. aeruginosa* strains highlights the critical need for alternative treatment methods [5]. The growing antibiotic resistance of *P. aeruginosa* is a significant clinical issue [6]. Utilizing multiple mechanisms, such as efflux pumps, impermeable membranes, and beta-lactamase production, the bacterium exhibits exceptional resistance to antibacterial agents. This resistance complicates treatment regimens and underscores the critical importance of exploring novel avenues for combating *P. aeruginosa* infections [7].

With antibiotic resistance on the rise, antibacterial peptides (AMPs) have emerged as potential replacements for antibiotics [8]. The naturally occurring AMPs, with potent antibacterial effects, are crucial components of the innate immune system in different organisms [9]. These peptides are active against many bacteria, including Gram-negative strains such as *P. aeruginosa*. AMPs primarily aim to disrupt bacterial membranes, resulting in cellular damage and frequently avoiding known resistance mechanisms. Due to their distinctive mechanism of functioning, AMPs are an alluring alternative for battling multidrug-resistant microbes [10]. The potential of AMPs to overcome *P. aeruginosa* resistance mechanisms necessitates their exploration as therapeutic options. By targeting bacterial membranes and disrupting essential cellular processes, AMPs can offer an effective strategy against strains that have developed resistance to traditional antibiotics [11]. The natural abundance of AMPs offers the potential for discovering new compounds with increased specificity and efficacy against *P. aeruginosa*, thanks to the diversity of AMPs in nature [12].

This study aimed to create new AMPs customized against *P. aeruginosa*, with a focus on strains resistant to conventional antibiotics. We evaluated the effectiveness of these peptides individually and in conjunction with standard antibiotics toward antibiotic-resistant and susceptible strains of *P. aeruginosa*. This research advances our knowledge of AMP effectiveness against *P. aeruginosa* while proposing potential novel therapeutic approaches for combating antibiotic resistance.

## Materials and Methods

### Ethical approval

Ethical approval was not required for this study.

### Study period and location

The study was conducted in December 2023 at the experimental base of the University of Jordan.

### Peptide design and synthesis

**Figure-1:**
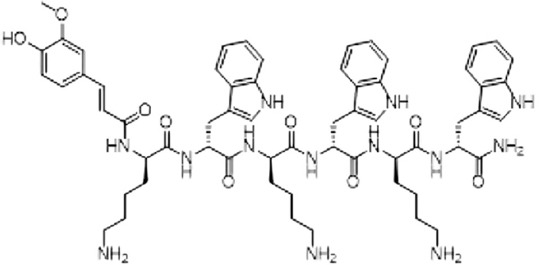
The structure of KW-23 peptide.

The peptide used in this study was obtained from GL Biochem Ltd., Shanghai, China, in a freeze-dried state following production through the solid-phase method. KW-23, a hex ultrashort AMP comprising six alternating subunits of lysine and tryptophan, is conjugated with hydrophobic ferulic acid moieties. The peptide was purified using reverse-phase high-performance liquid chromatography with a C18 Internsil® column (Thermo Fisher, USA). The octadecylsilyl groups column was eluted using an acetonitrile/H_2_O-trifluoroacetic acid gradient at a flow rate of 1.0 mL/min. Electrospray ionization-mass spectrometry confirmed the purification and identification of the synthesized peptide [13]. By employing the two positively charged amino acids [14], the design technique lowered the peptide’s cationicity, enabling electrostatic contact with bacterial cells’ negatively charged membranes. The peptide was conjugated with ferulic acid to confer the hydrophobicity necessary for membrane permeabilization. This molecule, highly hydrophobic in nature, functions as an anchor for peptide-membrane hydrophobic interactions. KW-23 has a net charge of +3 and a molecular weight of 1136.34 Da. Figure-1 illustrates the KW-23 peptide’s structure.

### Determination of minimum inhibitory concentrations (MICs) and minimum bactericidal concentrations (MBCs) for KW-23 and conventional Antibiotics

The MIC and MBC values for KW-23 and conventional antibiotics such as levofloxacin and chloramphenicol were measured. These antibiotics-rifampicin, amoxicillin, clarithromycin, doxycycline, vancomycin, cefixime, and gentamicin-were tested against both strains of *P. aeruginosa* using the microbroth dilution method as per Clinical and Laboratory Standards Institute (CLSI) guidelines with sterile 96-well polypropylene microtiter plates [15]. In Mueller Hinton broth (MHB), various bacterial strains resuscitated from frozen glycerol were cultured. Overnight, bacterial cultures in MHB were diluted to 10^6^ colony-forming unit (CFU)/mL. 50 μL of each peptide and 50 μL of the diluted bacterial suspension were added to separate wells of 96-well microtiter plates containing different concentrations of KW-23. Each well held a replicate of six distinct peptide concentrations. The enzyme-linked immunosorbent assay plate reader (Biotech, USA) was used to determine bacterial growth after an 18-h incubation at 37°C by measuring optical density (OD) at λ = 570 nm. The MIC of the antibacterial agent was identified. Each plate contained a positive control with 50 μL bacterial suspension and 50 μL MHB, and a negative control with 100 μL MHB. 10 μL from clear negative and previous turbid positive wells were streaked on sterile nutrient media agar. The MBC value represents the lowest concentration that kills 99.9% of bacteria after a 24-h incubation at 37°C. 15. The MICs and MBCs for each antibiotic were determined using the same method [15]. To guarantee the results’ reliability, all experiments were carried out in threes.

### Hemolytic activity of KW-23

The hemolytic activity of KW-23 against normal erythrocytes was measured according to Salama [16]. To ensure the experiment’s robustness and reliability, each one was carried out 3 times. The hemolysis equation for analysis is as follows:


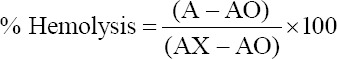


Where A: Is OD 450 with the peptide solution,

A0: Is the OD 450 of the blank.

And AX: Is OD 450 of the control (0.1% Triton X-100).

### 3-(4,5-dimethylthiazolyl-2)-2,5-diphenyltetrazolium bromide (MTT) cell proliferation assay

In this investigation, we utilized the Vero cell line procured from ATCC (CCL81), a commercially available mammalian cell line. MTT transforms into purple formazan following reduction within the cell by reductase enzymes metabolically active cells catalyze the reaction, generating purple formazan crystals. These purple crystals can be dissolved in dimethyl sulfoxide (DMSO), despite their insolubility in water. Spectrophotometry (Thermo Scientific, USA) measured the generated color of these crystals at a wavelength of 550 nm. 5 × 10^3^ cells were seeded in a flat-bottomed 96-well plate for the MTT assay and incubated at 37°C with 5% CO_2_ for 18–24 h for attachment. The following day, the cells in the plates were treated with Roswell Park Memorial Institute (RPMI) media containing varying concentrations of two peptides, which were suspended at 2, 4, 6, 8, and 10 mg/mL, and loaded with 200, 400, 600, 800, and 1000 g/mL of these peptides. An untreated medium served as the control. The plates were incubated at 37°C with 5% CO_2_ for 24 h. 24 h later, each well received 20 μL of 2.5 mg/mL MTT solution and was incubated with 5% CO_2_ at 37°C for 2–5 h. The wells were emptied post-incubation. 100 μL of DMSO was added to each well and mixed to dissolve formazan crystals, resulting in a clear purple solution. The plates were then placed on an absorbance microplate reader (BioTek, Winooski, VT, USA) and the absorbance at 550 nm was measured [17].

### Time killing curve

These experiments followed the method outlined in the M26-A document of CLSI. The bacterial suspension of 5 × 10^5^ CFU/ml was cultured in broth for three tubes. The first and second tubes house the test solution at 0.25 × MIC and 1 × MIC concentrations, while the third serves as the growth control. The incubation was conducted for different time durations: 0, 4, 6, 8, 10, 12, and 24 h. The dead cells percentage was calculated in comparison to the growth control by counting the number of living cells (CFU/mL) with the agar plate method in each tube. In general, the bactericidal effect is obtained with a lethality percentage of 90% for 6 h, which is equivalent to 99.9% of the lethality for 24 h [18].

### Fractional inhibitory concentration index (FICI) determination of KW-23 along with antibiotics

The microbroth checkerboard assay was used to investigate the synergistic activities of KW-23 and antibiotics [19]. The MIC of the antibiotics and KW-23 combination was determined by conducting the procedure in the previous section and adding both agents. Each experiment was repeated 3 times. Synergism was assessed through FIC values calculated by dividing the individual inhibitory concentrations of each antifungal agent in a combination by their combination’s inhibitory concentration [20].

FICI = (MIC peptide in combination/MIC peptide alone) + (MIC fluconazole in combination/MIC fluconazole)

Synergistic (FIC ≤ 0.5), additive (FIC 0.5 < FIC ≤1), indifferent (1 < FIC ≤ 4), or antagonist (FIC > 4).

## Results

### Antibacterial activity of the peptide

Table-1 shows KW-23’s 4.5 μg/mL and 20 μg/mL activity against *P. aeruginosa* strains ATCC 9027 and BAA-2108, respectively. One is a control strain, while the other is MDR. The MBC and MIC values were identical for both bacterial strains. The MIC and MBC values of the eight distinct antibiotics are tabulated in Table-1.

**Table-1:** The MICs and MBCs (μg/mL) of KW-23 and the nine antibiotics against the tested bacterial strains.

Compound	MIC (MBC) (µg/mL)	

	Control *P. aeruginosa* (9027)	MDR *P. aeruginosa* (BAA-2108)
KW-23	4.5 (4.5)	20 (20)
Levofloxacin	9 (9)	30 (30)
Chloramphenicol	80 (105)	150 (220)
Rifampicin	15 (15)	50 (50)
Amoxicillin	25 (25)	200 (200)
Clarithromycin	125 (125)	125 (125)
Doxycycline	6 (25)	34 (45)
Vancomycin	200 (350)	260 (400)
Cefixime	6 (10)	82 (110)
Gentamicin	2 (2)	20 (20)

*P. aeruginosa=Pseudomonas aeruginosa*, MIC=Minimum inhibitory concentration, MBC=Minimum bactericidal concentrations, MDR=Multidrug resistant

### Hemolytic activity of the peptides

The hemolysis percentage of the peptides is displayed in Table-2.

**Table-2:** Hemolytic activity of KW-23 against human erythrocytes after 60 min’ incubation.

Concentration (µM)	Hemolysis %
5	0
10	0
20	0
40	0
60	0
80	1
100	3

The results were recorded at λ = 450 nm

### MTT cell proliferation

**Figure-2:**
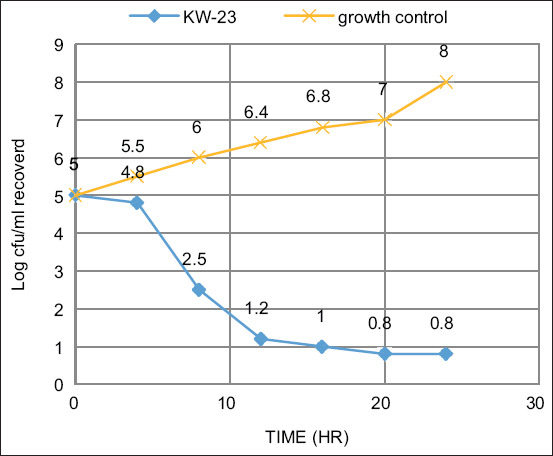
The 3-[4,5-dimethylthiazol-2-yl]-2,5 diphenyl tetrazolium bromide assay for the KW-23.

The half-maximal inhibitory concentration value for KW-23 in the cytotoxicity assay was 148 for the conjugate (Figure-2).

### Time-kill curve

**Figure-3:**
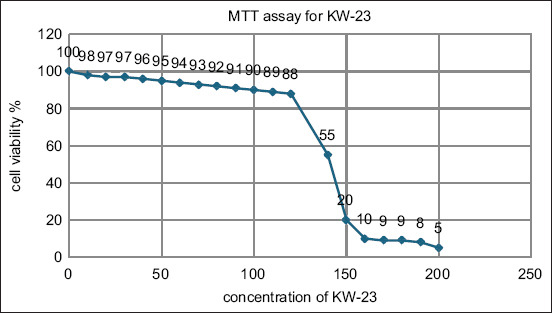
Time killing curve.

Figure-3 shows that KW-23 reduced the initial bacterial count by three logs.

### Antibacterial activity of KW-23 along with conventional antibiotics

The findings in Table-3 demonstrate the impact of various peptide KW-23/antibiotic combinations on both bacterial strains. In the combination of KW-23 and gentamicin, the most significant results were achieved against both control and MDR *P. aeruginosa* strains, with FICI values of 0.07 and 0.27, respectively.

**Table-3:** The checkerboard results of the effect of combinations of the peptide KW-23 with the antibiotics used in the study against the both bacterial strains used.

Bacteria strains	Antibiotics	MIC				FICI*

		Antibiotics	Antibiotics/KW-23	KW-23	KW-23/antibiotics	
Control *P. aeruginosa*	Levofloxacin	9	2	4.5	1.5	0.6
	Chloramphenicol	80	15	4.5	2	0.63
	Rifampicin	15	3	4.5	0.5	0.3
	Amoxicillin	25	10	4.5	0.25	0.45
	Clarithromycin	125	65	4.5	2	0.96
	Doxycycline	6	2	4.5	0.125	0.36
	Vancomycin	200	175	4.5	3.5	1.65
	Cefixime	6	0.5	4.5	1	0.3
	Gentamicin	2	0.125	4.5	0.025	0.07
MDR *P. aeruginosa*	Levofloxacin	30	15	20	10	1
	Chloramphenicol	150	60	20	15	1.15
	Rifampicin	50	20	20	5	0.65
	Amoxicillin	200	120	20	18	1.5
	Clarithromycin	125	80	20	5	0.9
	Doxycycline	34	10	20	2	0.4
	Vancomycin	260	150	20	15	1.3
	Cefixime	82	40	20	12	1.08
	Gentamicin	20	5	20	0.5	0.27

Synergistic (FIC ≤ 0.5), additive (FIC 0.5 < FIC ≤ 1), indifferent (1 < FIC ≤ 4), or antagonist (FIC > 4). FICI=Fractional inhibitory concentration index, *P. aeruginosa*=*Pseudomonas aeruginosa*, MIC=Minimum inhibitory concentration, MDR=Multidrug resistant

## Discussion

Due to the intrinsic antibiotic resistance of *P. aeruginosa* and its status as a global health crisis due to multidrug-resistant pathogens, innovative strategies are needed to combat this notorious opportunistic pathogen [21, 22]. Novel AMPs were synthesized, specifically targeting *P. aeruginosa* strains resistant to traditional antibiotics. AMPs, known for their broad-spectrum activity and ability to surpass resistance mechanisms, present an attractive option [23]. Safety concerns have largely hindered drug development for these peptides [24]. Based on the structure-function relationship insights of known AMPs [25], this study proposes a strategy for designing and synthesizing peptides. The ability of these peptides to treat both antibiotic-resistant and susceptible strains of *P. aeruginosa* was assessed. The novel peptide, KW-23, exhibits antibacterial activity similar to a traditional antibiotic while causing minimal harm to normal erythrocytes. KW-23’s MIC and MBC surpassed the antibiotics of diverse chemical classes against both strains. Both MIC and MBC values were identical, signifying bactericidal effect. The main cause of antibacterial activity in analog peptides lies in their ability to permeabilize bacterial cell membranes [26]. The KW-23 membrane-penetrating ability is most likely due to the use of charge segregation from the hydrophobic center [27]. The unspecified bacterial membrane disruption mechanism of the designed peptide is unlikely to trigger rapid resistance, thereby increasing its therapeutic duration. At concentrations up to 100 μg/mL, KW-23 does not cause human erythrocyte lysis, making it preferential for bacterial membranes over host cells. 6K-F17 selectively targets bacteria and is non-hemolytic to human erythrocytes, even in high concentrations above 500 μg/mL. Beaudoin *et al*. [28] found that electrostatic attraction to bacterial membranes increases the hydrophobicity of KW-23 beyond the threshold for spontaneous membrane penetration. The peptide demonstrated rapid bactericidal action against both strains within 15 min and continued effectiveness for up to 24 h, making it appealing as an antibacterial agent. In our study, the killing rate was akin to the kinetics observed with soluble peptides as observed by Aiemsaard *et al*. [29]. Release of ions and larger cellular molecules ensues when the peptide disrupts the cytoplasmic membrane [30]. The new compound KW-23 exhibits promising anti-MDR activity against *P. aeruginosa*. By decreasing individual drug dosages, delaying the development of resistance, and potentially eradicating resistant strains, combination therapy aims to minimize adverse effects [31]. The most effective outcomes were achieved when KW-23 was used in conjunction with gentamicin. Utilizing this combination may open up new treatment possibilities.

In *P. aeruginosa*, KW-23 in combination with doxycycline, chloramphenicol, and clarithromycin exhibited synergistic and partial synergy effects. Short peptides likely enhance antibiotic effectiveness by targeting the bacterial membrane. This effect is also attributed to the direct antibacterial activity of the peptide (KW-23) through interactions with intercellular targets, such as DNA, after entering the bacteria [32]. With amoxicillin, cefixime, rifampicin, and levofloxacin, synergy was demonstrated against the control strain, but not against MDR *P. aeruginosa*. This is expected, probably due to the presence of the AmpC β-lactamase and the MexAB-OprM efflux pump in this bacterium [33]. Within 2 h, KW-23 kills *P. aeruginosa* by disrupting its membranes and subsequently complexing with intracellular negatively charged components, including DNA and proteins [28]. Impairment of small molecule drug binding to targets by KW-23 could explain the lack of synergistic effects.

## Conclusion

Developing new AMPs against antibiotic-resistant *P. aeruginosa* is crucial for resolving the global antibiotic resistance issue. This study expands our knowledge of AMP’s actions and offers promising opportunities for new treatment strategies against multidrug-resistant bacteria. Synthesized peptides’ potential success could lead to novel clinical treatments, tackling antibiotic resistance. Future research should focus on developing new AMPs to combat antibiotic-resistant *P. aeruginosa*, leveraging the promising potential observed in this study. This study is limited by its primary focus on *in vitro* testing, necessitating further *in vivo* studies and consideration of resistance development and production feasibility for practical clinical application.

## Authors’ Contributions

RMD: Conceptualization and supervision. RMD and AHS: Investigation, methodology, and writing – review and editing. All authors have read, reviewed, and approved the final manuscript.
